# Estimating the Burden of Acute Gastrointestinal Illness in Grenada

**Published:** 2013-12

**Authors:** Lindonne M. Glasgow, Martin S. Forde, Samuel C. Antoine, Enrique Pérez, Lisa Indar

**Affiliations:** ^1^Department of Public Health and Preventive Medicine, St. George's University, St. George, Grenada; ^2^Ministry of Health, Ministerial Complex, St. George, Grenada; ^3^Pan American Health Organization, Panama; ^4^Caribbean Public Health Agency, 16-18 Jamaica Boulevard, Federation Park, Port of Spain, Trinidad and Tobago

**Keywords:** Acute gastroenteritis, Burden of Illness Study, Diarrhoea, Foodborne diseases, Foodborne pathogen, Laboratory survey, Population survey, Surveillance system, Grenada

## Abstract

This is the first study conducted in Grenada, with a population of approximately 108,000, to quantify the magnitude, distribution, and burden of self-reported acute gastroenteritis (AGE). A retrospective population survey was conducted in October 2008 and April 2009 and a laboratory survey from October 2008 to September 2009. The estimated monthly prevalence of AGE was 10.7% (95% CI 9.0-12.6; 1.4 episodes/person-year), with a median of 3 days of illness. Of those who reported AGE, 31% sought medical care (stool samples were requested from 12.5%); 10% took antibiotics; 45% took non-prescribed medication; and 81% reported restricted activity. Prevalence of AGE was significantly higher among children aged <5 years (23.5%, p<0.001). Of the AGE stool samples submitted to the laboratory for analysis, 12.1% were positive for a foodborne pathogen. *Salmonella enteritidis* was the most common foodborne pathogen associated with AGE-related illness. The estimated percentage of underreporting of syndromic AGE to the Ministry of Health was 69%. In addition, for every laboratory-confirmed foodborne/AGE pathogen, it was estimated that there were 316 additional cases occurring in the population. The minimum estimated cost associated with treatment for AGE was US$ 703,950 each year, showing that AGE has a potentially significant economic impact in Grenada.

## INTRODUCTION

Grenada is the main island of the Tri-island State comprising the mainland and two dependency islands Carriacou and Petite Martinique, with a total area of 133 square miles (344 sq. km) and a population of approximately 108,000. The main island—Grenada—is divided into six parishes: St. George, St. John, St. Marks, St. Patrick, St. Andrew, and St. David—with St. George parish being the most densely populated and St. Mark the least populated. While Grenada is characterized by lush tropical vegetation, the much smaller islands of Carriacou (area: 13 square miles, population~5,000) and Petite Martinique (area: 586 acres, population ~ 800) commonly experience harsher dry seasons, have no rivers, and surface-water is virtually non-existent. Tourism and agriculture are currently the main livelihood activities and sources of generating foreign exchange in the Tri-island State. The islands are still recovering from the effects of two devastating hurricanes—Ivan and Emily—which shattered the island in 2004 and 2005 respectively and severely damaged the agricultural sector and housing stock.

Acute gastroenteritis (AGE) and diarrhoea are common clinical outcomes of foodborne illness (1-3), which pose significant public-health problems, both in developed and developing countries (4-12). The World Health Organization (WHO) has found that diarrhoeal illness cause an estimated 1.87 million deaths globally (95% CI 1.56-2.19), accounting for approximately 19% of deaths in children aged <5 years (13). Diarrhoeal illnesses also have a huge negative impact on the tourism and agricultural sectors (6,12,14,15). Studies indicate that, in developing countries, children aged <5 years are at the greatest risk of AGE-related illness (13,16). Data from the Caribbean Epidemiology Centre (CAREC) indicate that AGE/diarrhoeal illnesses in the Caribbean region have increased from 60,574 cases in 2005 to 105,688 in 2006 (17). The ‘true’ burden of AGE in the region, however, remains unknown. Burden of Illness (BOI) studies have been proposed to estimate the community prevalence of diarrhoeal illnesses relating to food consumption, identify the sources of disease-causing organisms, and develop evidence-based policies for the control and prevention of foodborne diseases (12).

In all three islands—Grenada, Carriacou, and Petite Martinique—the Ministry of Health (MOH) has the responsibility for monitoring AGE-related illness. Healthcare services are provided through three hospitals, six health centres, and 30 health stations. Several private health clinics also operate in Grenada and Carriacou. The Pathology Laboratory at the General Hospital in Grenada is the main provider of laboratory services, although limited laboratory service is also provided by private facilities. The MOH conducts surveillance through collecting weekly reports of syndromic AGE from public healthcare facilities and aetiologic laboratory reports from the Pathology Laboratory. Limited data are also collected from private health facilities.

The Grenada National Strategic Plan for Health, 2007-2011 indicates that diarrhoeal illness is a major cause of morbidity in Grenada, with the highest rate of cases among children aged <5 years. In 2006, the MOH syndromic data showed 1,094 cases of AGE among children aged <5 years and 958 cases among persons aged >5 years (18). There are, however, limited data on the aetiology of AGE and the true burden of AGE-related illness in this island.

In 2007, St. George's University and the Ministry of Health in Grenada collaborated with CAREC and the Pan American Health Organization (PAHO) to conduct a BOI study in Grenada as part of the Caribbean Burden of Illness (BOI) Study. The goals of the Grenada BOI study were to estimate the prevalence and distribution of AGE, quantify the degree of underreporting of AGE in the population, identify key pathogens that cause AGE, estimate economic impact (burden) of AGE, and provide a better understanding of what the gaps in the surveillance system were. This was the first time that such a study was done in Grenada. Technical support and funding for this study were also provided by Teasdale-Corti grant programme of the Canadian Global Health Research Initiative (GHRI), a collaborative research funding partnership of the Canadian Institutes of Health Research, the Canadian International Development Agency, Health Canada, the International Development Research Centre, the Public Health Agency of Canada, and the Public Health Agency of Canada (PHAC).

## MATERIALS AND METHODS

The Grenada BOI study consisted of two core components: a population survey and a laboratory survey. The WHO definition for diarrhoea was used in defining an AGE case as being someone who had an acute (sudden) onset of diarrhoea, with or without fever (>38 °C or 100.4 °F), presenting with three or more loose or watery stools within the past 24 hours, with or without vomiting, and/or visible blood in the stool (19).

### Ethical approval

The St. George's University Institutional Review Board (IRB) and the MOH in Grenada granted ethical approval for the study (reference: 08010). Names of respondents were not included in the questionnaire. Respondents were informed of the purpose of the survey, and written consent was requested before the survey was administered.

### Population survey

**Figure 1.**
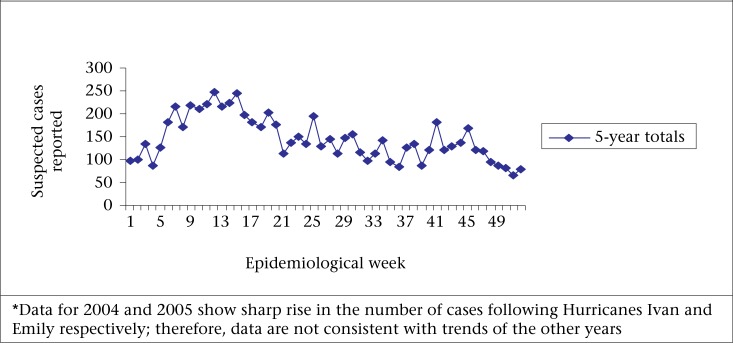
Total number of suspected cases of AGE reported to the National Surveillance Unit in Grenada for the period, 2001–2003 and 2006–2007*

A retrospective cross-sectional population survey was administered in two phases. The first survey was conducted during the 44-46 weeks in October 2008 to represent the low-AGE season, and the second survey was conducted during the 14-16 weeks in April 2009 to represent the high-AGE season. The low- and high-AGE seasons for Grenada were based on the trends of syndromic AGE over the period 2001-2003 and 2006-2007 (Figure 1). Trained interviewers administered the surveys via face-to-face interviews during evening hours and on weekends.

Using the 2006 estimated population of 108,000, a sample-size of 1,057 was calculated using Epi Info (version 6.0, Centers for Disease Control and Prevention, USA), with a 95% confidence interval, 50% prevalence, and 3% allowable error. An overall sample-size of 1,300 was used for this study, with 650 individual surveys conducted in each phase. Two hundred and sixty-two Enumeration Districts (EDs) were stratified by parish first, and the 1,300 surveys were proportionately distributed based on the number of households in each ED. A random numbers generator in Microsoft Excel (Microsoft Corporation, Redmond, WA) was used for generating household numbers for sampling in each ED. Each parish, along with Carriacou, was designated as a health region. Petite Martinique was included as an ED of Carriacou. The routes for survey administration were marked on ED maps provided by the Central Statistics Office (CSO) in Grenada. A standardized questionnaire developed by the Caribbean BOI steering team was slightly modified and used as the population survey instrument. A number was assigned to each questionnaire and was used for identifying the respondent in the database.

Persons who met the criteria (with the next birthday falling before the day of the survey) were selected for interview in the sampled households. Written consent was required for the interview. The neighbouring house was selected if any individual declined or was not available after 3 attempts to conduct the interview. If the selected individuals were aged <12 years, consent was required from their parents or guardians who answered the questions on their behalf. If the selected individuals were between 12 and 17 years of age, consent was required from the parents or guardians, and the selected individual answered the survey questions. Persons who were aged >18 years were eligible to give consent and also answer the survey questions. Individuals who were aged <1 year, unwilling or unable to participate in the survey, not physically present in the country at the time of survey, were <18 years old but did not receive parental consent, those who were prisoners, residents in hotels, guesthouses and inns, and were mentally disabled, were excluded from this study.

Respondents were asked if they had experienced diarrhoea in the past 4 weeks before the interview. Additional questions were included pertaining to sociodemographic and economic factors, secondary symptoms, healthcare-seeking behaviours, specimen submissions, use of medications, impact of illness, perceived cause of illness, and hygiene practices. Incentives, monetary or others, were not provided for participation in this survey.

### Estimation of the burden and underreporting of AGE

**Figure 2.**
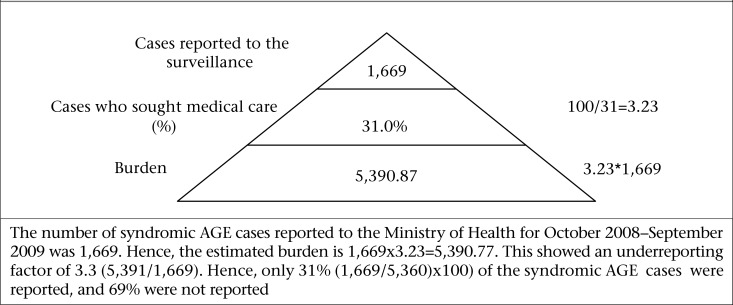
Estimation of underreporting and the burden of syndromic acute gastrointestinal illness in Grenada, 2008–2009

**Figure 3.**
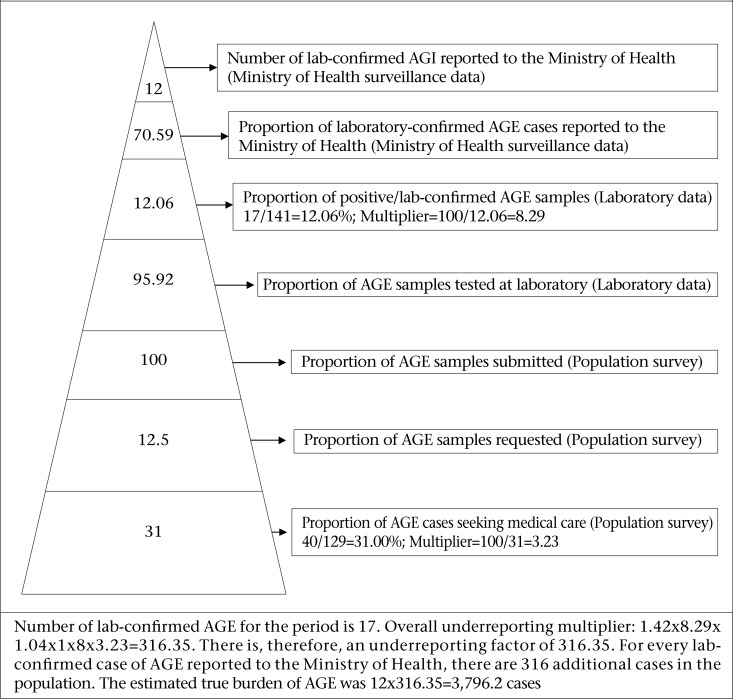
Estimation of the burden and underreporting of laboratory-confirmed AGE

The burden and the level of underreporting syndromic AGE and laboratory-confirmed AGE/foodborne diseases were calculated by comparison of the syndromic and laboratory-confirmed AGE data reported to the Ministry of Health for the period October 2008–September 2009 with that collected through the population and laboratory surveys. For syndromic AGE, the BOI pyramid was defined using the percentage of self-reported cases who sought medical care to estimate underreporting relative to syndromic AGE (Figure 2). For laboratory-confirmed AGE/foodborne pathogens, the BOI pyramid was defined using the percentage of AGE cases who sought medical care and submitted stool samples, the percentage of stool samples tested, samples testing positive for foodborne pathogen, and reported to the national surveillance. These proportions were then used in calculating multipliers for each level, and these multipliers were then used in estimating the underreporting of laboratory-confirmed AGE (Figure 3).

### Estimation of economic burden of AGE

Economic burdens of AGE estimates were calculated to assess the potential economic burden on patients associated with accessing healthcare and the treatment for AGE by multiplying the estimated episodes in the population per year with the cost of treatment.

### Laboratory survey

The laboratory survey was conducted from October 2008 to September 2009 at the Pathology Laboratory which is the primary laboratory that conducts microbial analysis for public and private health facilities in Grenada. All stool samples were transported to the Pathology Laboratory within 4 hours after collection and processed for selected foodborne pathogens within 2 hours after receipt. Of the stool samples submitted to the Pathology Laboratory from patients with a history of AGE, 96% were tested for *Salmonella*, *Campylobacter*, and *Shigella*, and 11% were tested for norovirus. Stool samples were usually processed within 2 hours after receipt at the laboratory. Standard cultural identification methods were used for processing the stool samples for *Salmonella*, *Campylobacter*, and *Shigella* bacterial pathogens as are used in routine diagnostic laboratories, using selective and enrichment culture techniques; a commercial ELISA kit (IDEIA Norovirus Elisa Kit) was used for detection of norovirus.

### Statistical analysis

Data were manually entered into a database created in EpiData (Lauritsen JM & Bruus M. EpiData, Version 3) and analyzed using Epi Info (version 6.0), using a 95% CI. Two-sample Independent *t*-tests were conducted to analyze statistically significant differences in the results.

## RESULTS

### Response rate and characteristics of respondents

A total of 1,300 surveys were attempted but 1,232 (94.8%) individuals were successfully contacted and invited to participate in the survey. Of these, 25 surveys were rejected due to incompleteness or inclusion of ineligible persons, leaving a total of 1,207 (92.8%) surveys which were used in the analysis—594 in Phase 1 (October 2008) and 613 in Phase 2 (April 2009). Comparison of the demographic profiles of survey respondents with residents in the general population indicated that there was overrepresentation of individuals aged 15-24 years (survey 27.5%, population 18.2%) and females (survey 55.9%, population 50.2%), and an underrepresentation of individuals aged 5-14 years (survey 11.9%, population 23.0%) in this study.

### Magnitude and distribution of self-reported cases of AGE

A total of 129 persons (10.7%) (95% CI 9.0-12.6) reported that they had sudden onset of diarrhoea with three or more watery or loose stools within 24-hour period with or without fever, dehydration, vomiting, or visible blood in the stool within 4 weeks prior to the interview. They were, therefore, classified as self-reported AGE cases. Using the calculated AGE prevalence of 10.7% and the formula outlined in the Appendix, the rate per person-year was calculated to be 1.4 episodes per person-per year. A similar monthly prevalence was observed for the identified low- and high-AGE seasons (low-AGE season: 10.6%; high-AGE season: 10.8%). Table 1 provides information on the sociodemographic characteristics of the self-reported cases in this study.

The highest monthly prevalence of AGE was found among children aged <5 years (23.5%), followed by those between 5 and 14 years of age (14.6%). The lowest reported prevalence of AGE was found among persons aged 45-64 years (5.7%) and ≥65 years (5.2%). AGE among children aged <5 years statistically differed from all other age-groups (p<0.001-0.003), except those between 5 and 14 years (p=0.113). The difference in prevalence between females (11.7%) and males (9.4%) was not statistically significant.

By health region, the highest monthly prevalence of self-reported cases was found in St. John (17.4%), which was significantly higher than the proportion of self-reported cases found in St. Patrick and St. Andrew (p=0.018 and p<0.001 respectively); the lowest monthly prevalence was found in St. Patrick (4.3%), St. David (9.1%), and St. Andrew (9.8%). Table 2 provides information on the distribution of self-reported cases of AGE in the health regions in Grenada.

The majority of self-reported AGE cases (66.0%) attributed their illness to consumption of food or drink. About half (54.3%) of the respondents reported that they washed hands either always or sometimes before eating meals, and 43.9% used soap. Three-quarters (75.2%) of respondents self-reported that they washed hands either sometimes or always after using the toilet, and 61.4% self-reported that they used soap to wash hands after using toilet.

While almost all respondents living on the mainland Grenada use pipe-supplied chlorinated water as their main source of drinking-water, all respondents from Health Region 7—Carriacou and Petite Martinique—used harvested rainwater as their main source of drinking-water. Of those living in Carriacou and Petite Martinique, 58.2% treated the water before drinking it. The most common treatment methods used were boiling (65.2%) and adding chlorine tablets/bleach (19.6%). There was no significant association between drinking-water source and water treatment with being a case of AGE (p=0.37).

**Table 1.** Sociodemographic characteristics of self-reported AGE cases (n=129) in Grenada

Variable	Residents (N)	Respondents (n)	Number and percentage of AGE cases	95% Confidence interval
Gender (n=1,207; p=0.20)				
Male	54,481	532	50 (9.4)	7.1-12.3
Female	52,151	675	79 (11.7)	9.4-14.4
Age (completed years) (n=1,207; p<0.001)				
1-4	9,948	81	19 (23.5)	14.8-34.2
5-14	20,189	144	21 (14.6)	9.3-21.4
15-24	20,624	332	36 (10.8)	7.8-14.8
25-44	27,938	322	35 (10.9)	7.8-14.9
45-64	17,485	212	12 (5.7)	3.0-9.7
≥65	6,448	116	6 (5.2)	1.9-10.9
Cultural group (n=1,207; p=0.86)				
African/Black	NA	1,087	116 (10.7)	8.9-12.7
Indian	NA	78	10 (12.8)	6.3-22.3
Asian	NA	0	0 (0)	0.0-84.2
European	NA	3	0 (0)	0.0-7.0
South American	NA	3	0 (0)	0.0-70.8
North American	NA	4	1 (25.0)	0.6-80.6
Monthly income (EC$) (n=900; p=0.18)				
Low income (0-1,000)	NA	345	42 (12.2)	9.0-16.2
Medium income (1,001-2,000)	NA	343	47 (13.7)	10.3-17.9
High income (>2,000)	NA	212	18 (8.5)	5.1-13.1
Education of mother (n=1,206; p=0.57)				
Primary	NA	414	38 (9.2)	6.7-12.5
Secondary	NA	474	59 (12.4)	9.7-15.8
Certificate/Diploma	NA	122	16 (13.1)	7.7-20.4
Undergraduate/Graduate	NA	35	3 (8.6)	1.8-23.1
Postgraduate	NA	14	0 (0)	0.0-23.2
Education of father (n=1,206; p=0.30)				
Primary	NA	408	36 (8.8)	6.8-12.1
Secondary	NA	330	46 (13.9)	10.5-18.3
Certificate/Diploma	NA	85	13 (15.3)	8.4-24.7
Undergraduate/Graduate	NA	25	2 (8.0)	1.0-26.0
Postgraduate	NA	15	1 (6.7)	0.2-31.9
Health region (HR)/Parish				
HR 1/St. Andrew	26,435	285	28 (9.8)	6.9-13.9
HR 2/St. Patrick	10,461	138	6 (4.3)	1.6-9.2
HR 3/St. George	37,403	421	49 (11.6)	8.8-15.2
HR 4/St. Mark	4,346	49	7 (14.3)	5.9-27.2
HR 5/St. John	8,405	92	16 (17.4)	10.3-26.7
HR 6/St. David	12,859	143	13 (9.1)	4.9-15.0
Carriacou and Petite Martinique	5,633	79	10 (12.7)	6.2-22.0

NA=Not available

### Symptoms and severity

The median episodes of diarrhoea among self-reported AGE cases in 24 hours was 4 (minimum 3, maximum 10), the number of days with AGE was 3 (minimum 1, maximum 14), and days spent at home without doing routine activities due to the illness was 2 (minimum 1, maximum 14). Of the 129 cases, 83.7% said they experienced abdominal pain, 35.7% experienced vomiting, 27.1% experienced headache, and 29.5% experienced nausea. Table 3 provides information on symptoms experienced by self-reported AGE cases.

### Healthcare-seeking behaviours and treatments

Of the 129 self-reported AGE cases, 40 (31%) reported seeking medical care, of whom only 12.5% were asked by their physicians to submit a stool sample. Antibiotics were prescribed for 10.0%; oral rehydration fluid, which is the WHO-recommended therapy for persons with AGE, was prescribed for 65.0%. Of those who received prescription for medication, 94.4% took the medication as prescribed by the physicians. Almost half of the self-reported cases (45.0%) reported using non–prescribed treatments for the diarrhoeal illness; of them, 19.1% used bush medicine, i.e. any part of a plant used for medicinal purposes without a physician's prescription, and 23.6% used oral rehydration fluid. Table 4 provides information about healthcare-seeking behaviours of the self-reported AGE cases.

**Table 2.** Distribution of self-reported cases of AGE by health region in Grenada

Health region/parish	Overall number and percentage of cases per health region/parish	95% CI
Health Region 1: St. Andrew	28 (9.8)	6.9-13.9
Health Region 2: St. Patrick	6 (4.3)	1.6-9.2
Health Region 3: St. George	49 (11.6)	8.8-15.2
Health Region 4: St. Mark	7 (14.3)	5.9-27.2
Health Region 5: St. John	16 (17.4)	10.3-26.7
Health Region 6: St. David	13 (9.1)	4.9-15.0
Carriacou and Petite Martinique	10 (12.7)	6.2-22.0

**Table 3.** Secondary symptoms experienced by self-reported AGE cases (n=129) in Grenada

Secondary symptom experienced by self-reported cases of AGE in Grenada	%	95% CI
Headache	27.1	19.7-35.7
Fever (measured)	16.7	15.0-31.2
Fever (not measured)	23.2	10.6-24.3
Nausea	29.5	21.8-38.1
Vomiting	35.7	27.4-44.6
Runny nose	7.0	3.2-12.8
Abdominal pain	83.7	76.2-89.6
Bloody diarrhoea	3.1	0.9-7.7
Cough	18.6	12.3-26.4
Sneezing	10.1	5.5-16.6
Sore throat	11.6	6.7-18.5

### Laboratory diagnosis of selected foodborne pathogens in stool samples

From 1 October 2009 to 30 September 2009, 147 stool samples were submitted to the Pathology Laboratory from patients with history of diarrhoeal illness, of which 141 (96%) were analyzed for foodborne pathogens–*Salmonella, Shigella, Campylobacter*, and norovirus. Overall, 12.6% tested positive for a foodborne pathogen. *Salmonella* (9.2%) and norovirus (1.4%) were the most commonly-identified pathogens.

### Estimation of underreporting of AGE to the MOH Surveillance Unit

Figure 2 and 3 show the surveillance pyramids defined to calculate the burden of illness for syndromic AGE and laboratory-confirmed AGE (for foodborne pathogens) in Grenada. Table 5 contains information required to calculate the burden of syndromic AGE and laboratory-confirmed AGE/ foodborne pathogens.

**Table 4.** Healthcare-seeking practices among self-reported cases of AGE in Grenada

Healthcare-seeking behaviour	%	95% CI
Sought medical care (n=129)	31.0	23.2-39.7
Was asked to submit specimen (n=40)	12.5	4.2-26.8
Submitted specimen upon request (n=5)	100	100.0-100.0
Took antibiotics (prescribed) (n=40)	10	8.0-12.0
Took oral rehydration fluid (prescribed) (n=32)	65.6	46.8-81.4
Took non-prescribed medications (n=129)	45.0	36.2-54.0

**Table 5.** Information for calculation of the burden of acute gastrointestinal illness in Grenada, October 2008–September 2009

Information	Source of data	Formula and value
Number of syndromic AGE reported to the Ministry of Health for the period October 2008–September 2009	Ministry of Health Surveillance Unit	1,669
Proportion of laboratory-confirmed AGE cases reported to the Ministry of Health for the period October 2008–September 2009	Ministry of Health Surveillance Unit	Laboratory-confirmed/positive AGE reported to the Ministry of Health= 80.0%. Total laboratory-confirmed AGE reported to the Ministry of Health =70.59% (12/17)
Number of lab-confirmed AGE cases actually isolated at the lab for the period October 2008–September 2009	Laboratory survey	Actual number of samples positive for AGE=17
Proportion of positive/laboratory-confirmed AGE (of AGE samples tested, what proportion was positive)	Laboratory survey	Number of samples positive for AGE/number of AGE samples tested=12.06% (17/141)
Proportion of AGE samples tested at laboratory (how often do the lab tests for a pathogen (at least 1 pathogen)	Laboratory survey	Number of samples tested for AGE/ number of samples received by lab =96% (141/147)
Proportion of physicians requesting AGE samples submitted to the laboratory	Population survey	Number of samples reported submitted/number of samples requested=100%
Proportion of AGE samples requested (of cases who sought medical care, proportion requested to submit sample)	Population survey	Number of samples requested/number of cases seeking medical care=12.5%
Proportion of AGE cases seeking medical care	Population survey	Number of AGE cases seeking medical care/number of AGE cases=31%
Number of AGE cases in population survey (meeting AGE case definition)	Population survey	Number of ill persons meeting AGE case definition=129

The estimated burden of syndromic AGE in Grenada for the one year period (September 2008–October 2009) was calculated as follows:

Number of syndromic AGE cases reported to the Ministry of Health for the specified period (1,669)x underreporting multiplier for seeking medical care (100/31 or 3.2)=5,390.80. This showed an underreporting factor of 3.3 (5,391/1,669). Hence, only 31% (1,669/5,360)*100) of the syndromic AGE cases were reported, and 69% were not reported (Figure 2).

The burden of laboratory-confirmed AGE/foodborne pathogens was calculated as follows:

A total of 129 AGE cases were identified among the 1,207 respondents who took part in the population survey. Of them, 40 (31%) sought medical care. From these 40 presenting cases, 5 (12.5%) were asked to submit stool samples, and all 5 complied. From the laboratory survey, a total of 147 stool samples were received, of which 141 (95.9%) were analyzed. Of these 141 analyzed samples, 17 (12.1%) contained an identified AGE pathogen but only 12 (70.6%) of these were reported to the Ministry of Health. Therefore, the overall underreporting multiplier factor for laboratory-confirmed AGE pathogens was 1.42×8.29×1.04×1×8×3.23=316.35. Thus, of the 12 laboratory-confirmed AGE cases that were reported to the MOH over the same period that the population survey was conducted, the estimated true burden of AGE was 12×316.35=3,796.2 cases (Figure 3).

### Socioeconomic costs

The estimated costs of seeking and receiving medical care for treating an AGE case in Grenada ranged from US$ 15 to 52, depending on the type of transportation, physician (public or private), laboratory service used (public or private), and medications prescribed. Using this range of costs, the annual economic burden on patients associated with the treatment for AGE was estimated to range from US$ 703,950 to 2,440,360. It should be noted that an estimate of the individual burden due to the lost quality of life is not included in the costs of socioeconomic burden. Additionally, the AGE cases who were unable to perform routine activities lost, on an average, between US$ 11 and 30 (minimum wage range/day) for each day that they were unable to attend work. Using the calculated median of 3 days without performing routine activities found in this study, the productivity losses potentially ranged from US$ 33 to 90 per person due to AGE-related illness.

## DISCUSSION

This study indicates that diarrhoeal illness is common in Grenada, with approximately 11% of the population being affected and an incidence rate of 1.4 episodes per person per year. This incidence rate was higher than that found in several developed countries, such as Canada (1.3 episodes per person-year for the period February 2001 to February 2002) (20), Ireland (0.60 episodes per person-year for the period December 2000 to November 2001) (4), and Australia (0.90 episodes per person-year for the period September 2001 to August 2002) (21). The prevalence of AGE in Grenada, however, is comparable with that found in Cuba (Grenada 10.7%, Cuba 10.6%) (22).

In Grenada, the prevalence of AGE among children aged <5 years was significantly higher than among other age-groups. This finding is consistent with that from other studies and the WHO statements of a global pattern that establishes AGE as highest among children aged <5 years. The Ministry of Health (MOH) in Grenada has listed AGE-related illness as a common cause of morbidity in children on the island. Younger children are at higher risk for AGE as an outcome of their tendencies to practise poor hygiene. Results from the Global School Health Survey conducted in Grenada in the first half of 2008 showed that 11.9% of students in secondary schools never or rarely washed their hands before eating; 3.7% also indicated that they never or rarely washed their hands after using a toilet or latrine (23). While these percentages represent a small number of children, disease-causing organisms can be transferred to a larger number of children and result in higher incidence of related illnesses. This study also found that the level of handwashing before meals and after using the toilet, particularly with soap, was not very high, which inadvertently contributes to the spread of disease-causing organisms.

In this study, a similar monthly prevalence for the low- and high-AGE seasons (10.6% and 10.8% respectively) was found, which might have been indicative of outbreaks occurring during the low season. Syndromic data for the period 2001-2003 and 2005-2007 also showed sporadic AGE peaks occurring during the low season. These outbreaks, which usually occur in isolated locations, should be carefully monitored by the MOH, and necessary controls and preventive responses should be implemented.

Harvested rainwater is the main source of drinking-water in Carriacou and Petite Martinique, and the dry season (high-AGE season) has been noted for increased diarrhoea and vomiting, particularly among children on this small island. The outbreaks are assumed to be associated with water being drawn from low-level cisterns, which may contain higher concentrations of microbial contaminants. The highest reported rates of AGE in Carriacou and Petite Martinique typically corresponded with the harshest and the longest dry seasons. In this study, however, a lower proportion of self-reported AGE cases were found in Carriacou and Petite Martinique during the dry season than in the wet season. This finding requires further investigation to determine how interactions between environmental and other factors, such as the condition and treatment protocols employed in harvested rainwater systems, contribute to or reduce the incidence of AGE among residents on the islands.

While this study indicates that AGE is causing significant morbidity in Grenada, the use of homemade remedies to treat this illness may have likely contributed to the high levels of underreporting observed, resulting in further limitations on aetiology, trends, and impact of AGE at the national level. While the MOH has determined high- and low-AGE seasons in Grenada, this is the first study undertaken to obtain evidence of the likely burden of the disease in the Tri-island State. An underreporting factor of 316 for laboratory-confirmed foodborne AGE pathogens indicated that, for every case currently recorded, there were 316 additional cases occurring but not reported to the MOH Surveillance Unit. This high rate of underreporting highlights an urgent need to address the potentially huge burden of AGE in Grenada and the need for adjustments in medical practice and improvements in the surveillance system. Other notable findings from this study were the very low number of stool specimens requested by physicians to confirm AGE (12.5%) and the incomplete reporting of AGE cases by the laboratory to the MOH (only 70.59% of confirmed AGE cases were reported to the MOH). Furthermore, while there was a general increase in the number of samples submitted during the high-AGE season, non-adherence to sampling protocol by healthcare workers (for example, not storing samples at 4 °C during transportation and delivering samples 4 hours after collection) may have contributed to the proliferation of other organisms in the samples, besides the organism responsible for causing AGE, thereby affecting isolation in the laboratory.

The literature shows that AGE is closely associated with conditions that exist as a result of poverty. Additionally, urbanization has been identified as a key factor that may also contribute to the condition. Gouyave town of St. John parish is characterized by crowded living conditions, particularly in the centre of the town and along which a river called the Great River passes through. The Great River is used both as a dump as well as the main source of water for domestic and hygienic purposes for several households along the riverbanks. The town is also characterized by a large number of small, makeshift food outlets. Many of these outlets on the main streets are owned and operated by persons without food vendors’ license, which increases the likelihood of improper food handling. While many self-reported AGE cases attributed their illness to consumption of food and drink (66.0%), the questionnaire did not ask respondents if the food they consumed was prepared domestically or outside the home. Further research may be useful to compare between the risk associated with consumption of foods prepared at home and those purchased from outside the home.

### Limitations

There were several limitations in this study. There was an overrepresentation of females and persons aged 15-24 years and underrepresentation of persons aged 5-14 years in this study. These biases may be due to practices of convenient sampling by surveyors. To minimize this from happening, surveyors were re-trained before conducting the second phase of the survey. Given that the respondents were asked to recollect if they had any diarrhoeal illness that matched our AGE definition over the past four weeks, it is unlikely that many would have forgotten this for the severity of typical AGE symptoms. Also, as is expected in retrospective studies, the potential of recall bias cannot be ruled out. Recall bias is a limitation of retrospective studies. Retrospective methods may be subject to more recall bias than prospective studies. Nevertheless, similar methods to other retrospective studies were used, thereby enabling comparisons between studies.

### Conclusions and recommendations

Diarrhoeal illness is common in Grenada, with approximately 11% of the population being affected. *Salmonella enteritidis* is the most common pathogen associated with the illness, which also has major economic impact. Underreporting is high, and there is a need to improve the surveillance system to monitor and control AGE in Grenada.

Some recommendations which may be useful for monitoring and controlling AGE in Grenada are: (i) introducing public education programmes which promote and encourage proper hygiene practices; (ii) implementing more robust surveillance systems of street-based food vendors; and (iii) strengthening of the overall quality control monitoring of the farm-to-table food production and preparation continuum. Further, improved collaboration between the MOH and other ministries and organizations with the responsibility for food safety and the environment is essential to strengthen capacity, improve surveillance systems, and ensure that appropriate information is tabled for consideration in the development and implementation of policies that address control and prevention of foodborne diseases.

Strengthening laboratory capacity to test for a wider range of organisms will also assist in identifying which pathogens are of main concern and, hence, need attention in controlling AGE in Grenada. The results from this study indicate the high likelihood that patients will comply with physicians’ instructions to submit a stool sample for analysis and, therefore, this can be used as an opportunity to encourage more physicians to ask their suspected AGE patients to submit stool samples for laboratory analysis. Further, improved adherence to sample collection and storage protocols will increase the laboratory's chances of correctly isolating the organisms in stool samples. Finally, measures should be taken to ensure consistent and regular reporting to the national and regional surveillance units for timely identification of outbreaks and implementation of control measures.

## ACKNOWLEDGEMENTS

The authors wish to acknowledge all the institutions and persons who contributed to this study. In particular, we are especially grateful to Teasdale-Corti grant programme of the Canadian Global Health Research Institute (GHRI), Caribbean Epidemiology Centre (CAREC), and PAHO for providing the funds and technical support to undertake this study. The authors are also grateful for the support and help of the Ministry of Health in Grenada, Department of Public Health and Preventive Medicine at St. George's University, the Windward Island Research Foundation (WINDREF), and the Public Health Agency of Canada for providing technical and logistic support.

## Figures and Tables

**Appendix.**
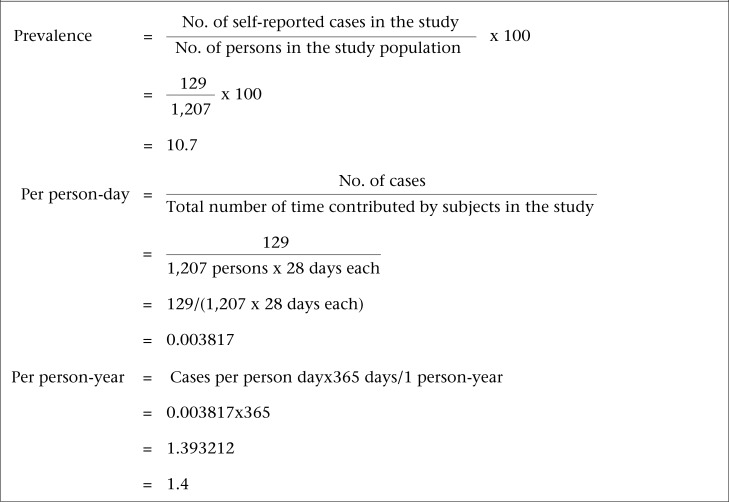
Formula used in calculating prevalence and rate per person-year in the burden of acute gastrointestinal illness study in Grenada
